# 3D-Printed Capacitive Sensor Objects for Object Recognition Assays

**DOI:** 10.1523/ENEURO.0310-20.2020

**Published:** 2021-01-25

**Authors:** Kasey P. Spry, Sydney A. Fry, Jemma M.S. DeFillip, S. Griffin Drye, Korey D. Stevanovic, James Hunnicutt, Briana J. Bernstein, Eric E. Thompson, Jesse D. Cushman

**Affiliations:** 1 North Carolina State University, Raleigh, NC 27695; 2 Neurobehavioral Core Laboratory, National Institute of Environmental Health Sciences, National Institutes of Health, Durham, NC 27709; 3 Fabrication and Repair Studio, National Institute of Environmental Health Sciences, National Institutes of Health, Durham, NC 27709

**Keywords:** 3D printing, capacitive, object recognition, open source, rodent

## Abstract

Object recognition tasks are widely used assays for studying learning and memory in rodents. Object recognition typically involves familiarizing mice with a set of objects and then presenting a novel object or displacing an object to a novel location or context. Learning and memory are inferred by a relative increase in time investigating the novel/displaced object. These tasks are in widespread use, but there are many inconsistencies in the way they are conducted across labs. Two major contributors to this are the lack of consistency in the method of measuring object investigation and the lack of standardization of the objects that are used. Current video-based automated algorithms can often be unreliable whereas manual scoring of object investigation is time consuming, tedious, and more subjective. To resolve these issues, we sought to design and implement 3D-printed objects that can be standardized across labs and use capacitive sensing to measure object investigation. Using a 3D printer, conductive filament, and low-cost off-the-shelf components, we demonstrate that employing 3D-printed capacitive touch objects is a reliable and precise way to perform object recognition tasks. Ultimately, this approach will lead to increased standardization and consistency across labs, which will greatly improve basic and translational research into learning and memory mechanisms.

## Visual Abstract

**Figure:**
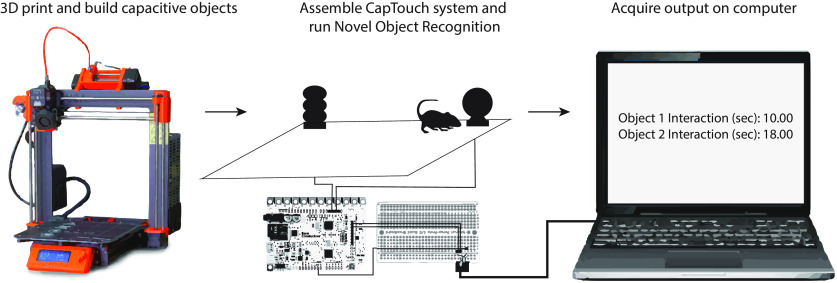

## Significance Statement

Object recognition assays are widely used in basic research and preclinical models; however, there is a profound lack of standardization in the objects used and scoring methods employed. Here, we show a proof-of-principle demonstration that employing 3D-printed capacitive objects is a cost-effective, reliable, and precise way to perform object recognition tasks when compared with manual scoring. This novel approach could ultimately contribute to a more standardized approach to object recognition tasks, which would greatly improve reliability in basic and applied neurobehavioral research.

## Introduction

Object recognition tasks are widely used assays for studying learning and memory in rodents (Ennaceur, 2010; Antunes and Biala, 2012; Heyser and Chemero, 2012; Lueptow, 2017). While a wide variety of protocols have been developed for novel object recognition (NOR), the task involves two basic phases: a *familiarization* phase where the animal becomes acquainted with the objects and a *test* phase where the original familiar objects are changed (typically replaced by a different object, or moved to a novel location or context). During familiarization, the animal encodes the object’s features, location, and context. During the test phase, because of the rodent’s natural innate preference for novelty, it should spend more time investigating the modified object(s) compared with the unmodified object(s). Intact learning and memory are inferred based on increased investigation of the modified object(s) during the test phase, i.e., we infer that the animal recognizes the object as novel and thereby directs greater investigative behavior toward it (Heyser and Chemero, 2012; Leger et al., 2013; Lueptow, 2017).

These tasks are in widespread use, but there are many inconsistencies in the way they are conducted. Two major issues are the lack of consistency in the method of measuring object investigation and the lack of standardization of the objects used. The main methods of scoring object recognition tasks are video-based automated software and manual scoring. Current video-based automated systems can often be unreliable, lack temporal precision, and can be costly, whereas manual scoring is time-consuming, tedious, and subjective. A lack of standardization of the objects used in object recognition is also a concern in object recognition tasks. Examples of objects used during object recognition tasks include plastic toys, glass bottles, stacking squares, and metal cans. Object properties can differ across a large number of dimensions such as shape, texture, color, material, reflectivity, and size (Bevins et al., 2002; Benice and Raber, 2008; Ennaceur, 2010; Antunes and Biala, 2012; Heyser and Chemero, 2012; Leger et al., 2013; Lueptow, 2017). These properties strongly influence the investigation of the objects, and different affordances offered by objects can strongly bias the results (Ennaceur, 2010). Despite such potential confounds, there has been little effort to develop a standardized approach for the selection of objects.

To resolve these issues, we sought to design and implement 3D-printed objects for object recognition tasks, so that objects can be standardized and reproduced across labs. In addition, to promote a more standardized method for measuring object investigation, we developed a capacitive touch sensing approach to quantify investigation using the Arduino-based MPR121 capacitive touch sensor controller, making the objects themselves the sensors. We used a 3D printer and low-cost off-the-shelf components to aid in widespread adoption and cross-lab validation. The objects were tested in object recognition tasks and compared with manual scoring. Two options for the Capacitive Touch (CapTouch) system were created. CapTouch 1.0, which used conductive filament, and CapTouch 2.0, which used traditional filament combined with copper tape inside the object. CapTouch 2.0 was created after CapTouch 1.0 to provide an additional lower-cost method of creating objects that also provides more options for the printing material. We provide details about the materials and build instructions needed for both iterations as well as the validation from both sets of experiments.

## Materials and Methods

### Components and construction

**Figure 1.**
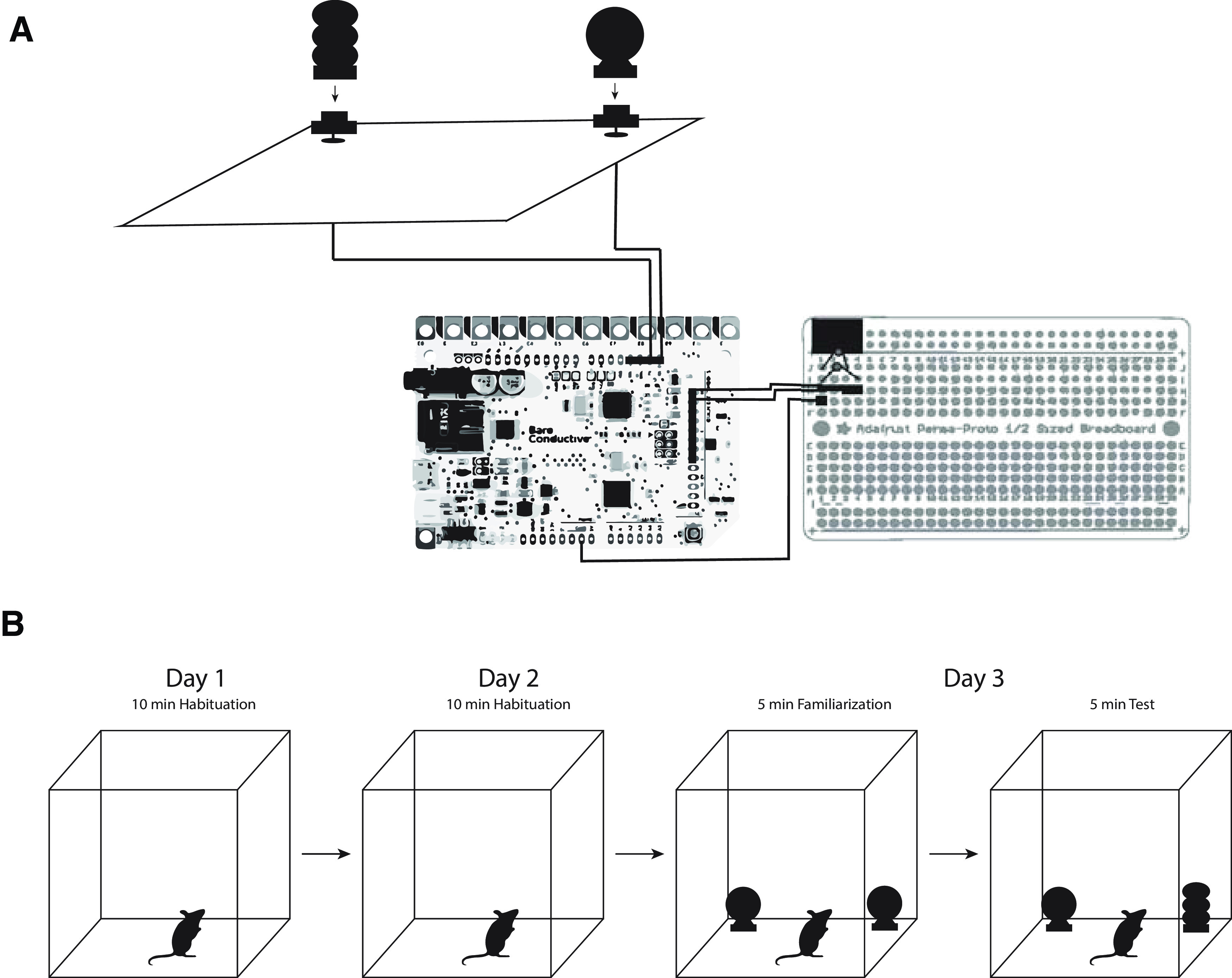
CapTouch overview. ***A***, Diagram of basic CapTouch system setup with Bare Conductive capacitive sensing board and breadboard. ***B***, Basic novel object recognition workflow.

A basic diagram for the system is shown in Figure 1*A*. All components needed for the two CapTouch systems are listed in Tables 1, 2, respectively. The estimated cost of making the CapTouch systems is about $85.00 (this does not include Noldus products and the cost of constructing the chambers used to run the experiments). Detailed build instructions will be made available on the NIEHS-nbc GitHub page. 3D models for the objects were designed using Blender 2.79, and Autodesk Netfab software was used to optimize the models before slicing. The final slicing and gcode generation were completed using PrusaSlicer and printed via Prusa i3 MK3s 3D printers, and this code will be available on the NIEHS-nbc GitHub page.

**Table 1:** Component list for CapTouch 1.0

Component	Quantity	Supplier	Part number	Price
Touch board	1	Bare Conductive	NA	$49.91 (list price: $62.39)
Electric paint 10 ml	1	Bare Conductive	NA	$11.04
Perma-proto half-sized breadboard PCB-single	1	Adafruit	1609	$4.50
Proto-pasta conductive PLA- 1.75 mm (0.5 kg)	1	MatterHackers	MUW33A27	$49.99 (list price: $56.00)
RJ45 8-pin connector	1 per touch board	SparkFun	PRT-00643	$1.50
Short headers kit for feather-12- pin + 16-pin female headers	2 per touch board	Adafruit	2940	$1.50
Solid-core wire spool-25 ft- 22AWG	1	Adafruit	290	$2.95
Magnet-1/2” diameter × 1/10” thick	1 per object	K&J Magnetics	D8H1	$0.83
Diffused 5-mm LED (25 pack)	1	Adafruit	299	$4.00
USB-IO box	1	Noldus	NA	$1535.00
Ethernet cable	1 per touch board	Adafruit	994	$2.75
USB cable-USB A to micro-B-3 foot long	1 per touch board	Adafruit	592	$2.95
Resistor-10 KΩ -pack of 25	2 per touch board	Adafruit	2784	$0.75

CapTouch 1.0 list of build components needed for the system. The component name, number needed, supplier, part number, and price are provided.

**Table 2:** Component list for CapTouch 2.0

Component	Quantity	Supplier	Part Number	Price
Flexfill 98A Powder Beige filament 500 g	1	Prusa Research	FLM-FLX-175-PBG-98A	$33.99
Flexfill 98A luminous green filament 500 g	1	Prusa Research	FLM-FLX-175-GRN-98A	$33.99
PETG Prusa orange filament 1 kg	1	Prusa Research	PRM-PETG-PRO-1000	$29.99
Touch board	1	Bare Conductive	NA	$49.91 (list price: $62.39)
Solid-core wire spool-25 ft- 22AWG	1	Adafruit	290	$2.95
Perma-proto half-sized breadboard PCB-single	1	Adafruit	1609	$4.50
RJ45 8-pin connector	1 per touch board	SparkFun	PRT-00643	$1.50
Short headers kit for feather-12-pin + 16-pin female headers	2 per touch board	Adafruit	2940	$1.50
Diffused 5-mm LED (25 pack)	1	Adafruit	299	$4.00
USB-IO box	1	Noldus	NA	$1535.00
Ethernet cable	1 per touch board	Adafruit	994	$2.75
USB cable-USB A to micro-B- 3 foot long	1 per touch board	Adafruit	592	$2.95
Resistor-10 KΩ	2 per touch board	Adafruit	2784	$0.75
Copper foil tape with conductive adhesive-25 mm × 15 m roll	1	Adafruit	1127	$19.95

CapTouch 2.0 list of build components needed for the system. The component name, number needed, supplier, part number, and price are provided.

**Figure 2.**
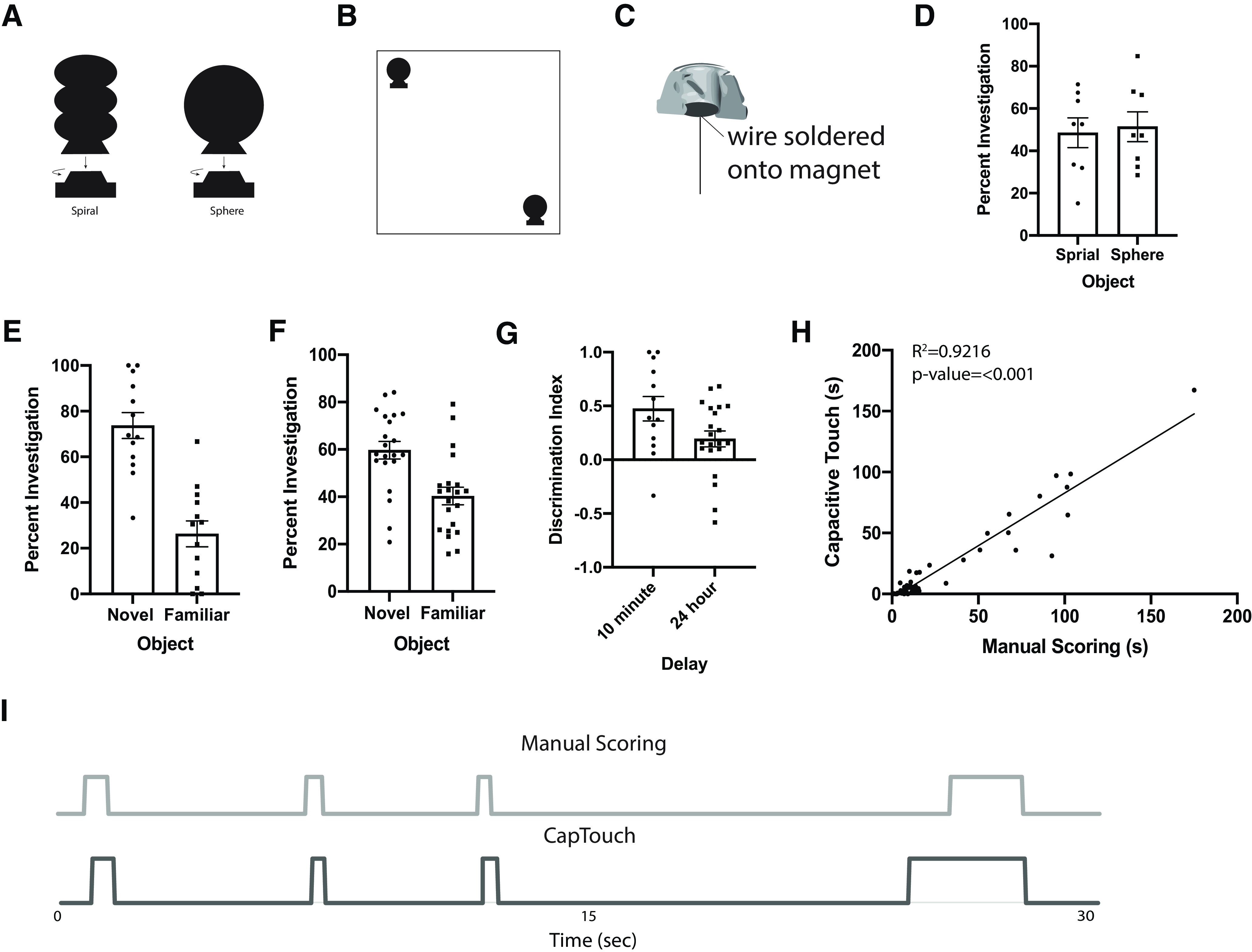
CapTouch 1.0 NOR results and validation. ***A***, Diagram of CapTouch 1.0 objects spiral (right) and sphere (left). ***B***, Object placement during NOR experiment. ***C***, Cross-sectional view and diagram of wire attachment to the base. ***D***, Percent investigation between the spiral and sphere object during the object preference test (±SEM). ***E***, Percent investigation between the novel and familiar object with a 10-min delay during the NOR experiment. Two mice were excluded from the novel object recognition results because of CapTouch sensing malfunction (±SEM). ***F***, Percent investigation between the novel and familiar object with a 24-h delay during the NOR experiment (±SEM). ***G***, Discrimination index comparing investigation between the novel and familiar objects for the 10-min delay (*p* = 0.0013 relative to chance) and the 24-h delay (*p* = 0.0165 relative to chance) NOR experiments. ***H***, Correlation of object investigation duration (seconds) during the object preference test and both the familiarization and test days of the NOR test between capacitive touch sensing and manual scoring (*R*^2^ = 0.9162, *p* < 0.001; see Extended Data Figure 2-1 for further validation with an additional manual scorer). ***I***, 30-s example of capacitive touch triggering compared with manual scoring. For additional validation analysis, see Extended Data Figure 2-2.

The CapTouch 1.0 objects and their bases were printed with conductive filament to record interactions. The design allowed for easy removal and attachment of the object to the base via a twist-off design. The objects for CapTouch 1.0 were connected to the Bare Conductive Touch Board using solid core wire which was attached to a magnet that was inserted in the objects on one end and connected to the header pins on the touch board on the other end (Fig. 2*C*).

10.1523/ENEURO.0310-20.2020.f2-1 Extended Data Figure 2-1 ***A***, Correlation of object investigation duration (seconds) between capacitive touch sensing and an additional manual scorer (*R*^2^ = 0.9167, *p* < 0.0001). ***B***, Comparison of manual scorers correlated against each other (*R*^2^ = 0.9950, *p* < 0.0001). Download Figure 2-1, PDF file.

10.1523/ENEURO.0310-20.2020.f2-2 Extended Data Figure 2-2 ***A***, Histogram of time between object interactions (inter-interaction interval) for capacitive sensing (mean = 13.9 ± 2.7s) and manual scoring (mean = 17.3 ± 1.7 s) for the CapTouch 1.0 experiments (i > 0.05, moody test). ***B***, Violin plot of interinteraction intervals for capacitive sensing and manual scoring for the CapTouch 1.0 experiments. Download Figure 2-2, PDF file.

**Figure 3.**
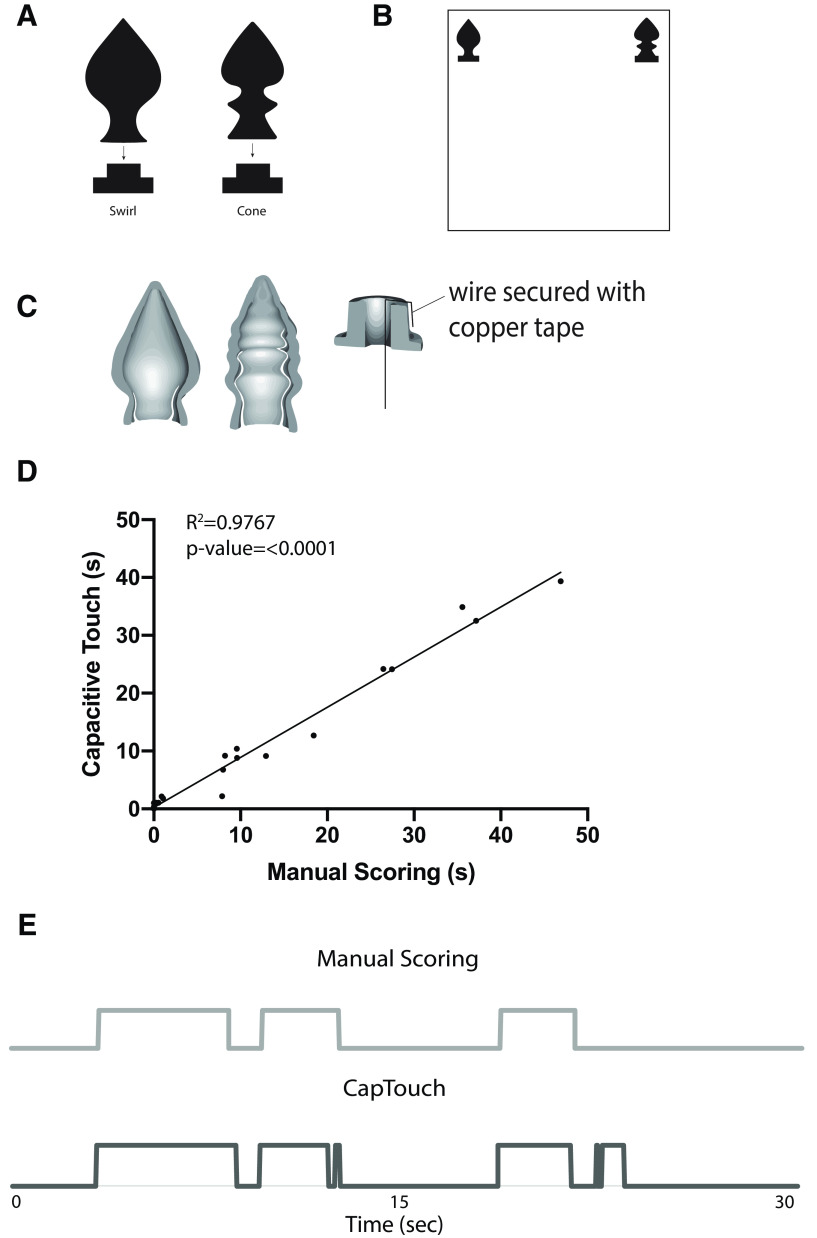
CapTouch 2.0 validation. ***A***, Diagram of CapTouch 2.0 objects swirl (left) and cone (right). ***B***, Object placement during system validation experiment. ***C***, Cross-sectional view of hollow objects and diagram of wire attached to base. ***D***, Correlation of object investigation duration (seconds) for both familiarization and test days between capacitive touch sensing and manual scoring (*R*^2^ = 0.9767, *p* < 0.0001; see Extended Data Figure 3-1 for validation of an additional manual scorer). Because of low interaction from some of the mice during the CapTouch 2.0 validation experiment, two highly interactive mice were run through an additional trial during the familiarization and test phases to acquire more interaction data. One object during a trial had a CapTouch sensing malfunction and was not included in the validation correlation. ***E***, 30-s example of capacitive touch triggering compared with manual scoring.

The CapTouch 2.0 objects were printed in a non-conductive Flexfill filament and their associated bases in a PET-G filament. The objects were hollow to allow strips of copper foil tape to be placed inside the objects to serve as the capacitive sensor. The objects were connected to the bases by a simple peg-in-hole design. Solid core wire which was attached at one end to the base by copper tape was connected to the header pins on the touch board on the other end (Fig. 3*C*). In both iterations, the CapTouch bases were connected to the arena floor using hot glue.

10.1523/ENEURO.0310-20.2020.f3-1 Extended Data Figure 3-1 ***A***, Correlation of object investigation duration (seconds) between capacitive touch sensing and an additional manual scorer (*R*^2^ = 0.9313, *p* < 0.0001). ***B***, Correlation of manual scorers against each other (*R*^2^ = 0.9642, *p* < 0.0001). Download Figure 3-1, PDF file.

The touch board from Bare Conductive is an Arduino Leonardo based ATmega32U4 microcontroller board that runs at 16 MHz from 5 V with capacitive touch and MP3 decoder ICs. It uses a MPR121 chip that gives it twelve capacitive touch/proximity sensing electrodes (Bare Conductive, 2018). Capacitive touch/proximity sensing electrodes are devices that can detect the presence or absence of an object by using a change in capacitance based on a change in the electrical field that is generated around the sensor. Capacitive touch/proximity sensors operate as a simple capacitor. The face of the object (the sensing face) is electrically connected to an internal oscillator circuit and the animal (the target) acts as the second plate of the capacitor which produces an electrostatic field. The external capacitance between the object and the animal forms part of the feedback capacitance of the oscillator circuit; when the animal approaches the sensor, the oscillations increase until the set threshold level is reached and activates an output. Capacitive touch/proximity sensors sensitivity can be adjusted which can change the operating distance to the target (Moermond, 2017). The MPR121 uses an auto-calibration mechanism that detects background capacitance (which varies as a function of the size of the object used for the sensor) and subtracts this to achieve an optimized baseline. The touch board can be programmed using the Arduino IDE and open source code was modified so that the touch/proximity threshold of the capacitive touch electrodes was set at 1 and the release threshold was at 2 (Bare Conductive, 2018; code available on the NIEHS-nbc GitHub). The Arduino IDE sketch was programmed to send a TTL pulse from a corresponding output pin when a touch electrode detected a touch. This TTL pulse was sent to a Noldus IO Box to be recorded by the analysis software, Ethovision. TTL signal could be read out via a variety of methods, however, we chose the Noldus IO Box as it would allow us to easily cross-validate with automated and manual scoring. Readout parameters included the total number of interactions, the amount of time of each interaction, and the total summed interaction time.

### Animals

Adult female and male C57BL/6 mice were obtained from Taconic Farms and were group-housed on a 12/12 h light/dark cycle. All experiments were performed during the dark phase. For the object preference test, four female and four male mice were used at three months of age. For the CapTouch 1.0 experiments, eight female and eight male mice were used at four months of age. For the CapTouch 2.0 experiments, four female and four male mice were used at three months of age. For the 24-h retention interval experiments using CapTouch 1.0 objects, two sets of eight female and eight male mice were used at three months of age. All animal procedures were performed in accordance with the NIEHS animal care committee’s regulations.

### Procedure

Before the novel object recognition task took place during the CapTouch 1.0 experiments, an object preference task was performed with naive mice to assess the preference or lack of preference for one of the objects over the other. This is a critical step in the novel object recognition task because it ensures that there is not an innate preference for one of the objects, which could skew results obtained from the novel object recognition task. This task occurs in two phases: habituation and test. During the habituation phase, the animal was placed in the middle of the open-field arena and was allowed to explore the open-field arena for 10 min. During the test phase, one of each object was placed in the corners of the arena, the animal is introduced into the middle of the arena and allowed to explore the arena and objects for 10 min. The objects were counterbalanced between the two arenas to account for side preference and they were placed directly in the corners against the walls. Duration of time each mouse spent investigating the objects was recorded and calculated to ensure there was no significant difference between the length of time spent investigating each object.

The novel object recognition task took place during three phases: habituation, familiarization, and test phase. In the habituation phase, the animal was allowed to explore a dimly lit (5–10 lux) open-field arena for 10 min on two consecutive days (20 min total). During the familiarization phase, two capacitive touch objects were set up in the arena. The animal was placed in the center of the arena and was allowed to explore the arena and objects for 10 min. After a retention interval of 10 min, the test phase occurred. During the test phase, one of the objects was switched for a novel object and the second object stayed the same as during the familiarization phase. The animal was placed in the center of the arena and was allowed to explore the arena and objects for 10 min.

A 24-h retention interval was also tested in the novel object recognition task as used in earlier reports (Pereira et al., 2014; Vogel-Ciernia and Wood, 2014; Kwapis et al., 2018; Tuscher et al., 2018). In attempt to increase object investigation, the mice were exposed to different objects with the same 3D-printed filament in their home cages before the familiarization and test phases for 10 min while being handled on the day before the experiments. During the familiarization phase, mice were exposed to objects until they interacted for a cumulative 30 s or remained in the arena to explore the objects for 30 min. Mice that did not reach ten cumulative seconds of investigation during the familiarization phase were not included in the test phase. During the test phase, mice were allowed to explore the arena and objects for 30 min. All objects were placed in the corners of the arena directly against the wall (Fig. 1*B* shows a basic workflow of the novel object recognition experiment). The novel and familiar objects were counterbalanced across mice. Objects were thoroughly cleaned with 70% ethanol between animals and allowed to dry and the arena was thoroughly cleaned with Windex between animals and allowed to dry. The Bare Conductive touch boards were reset before every trial to engage the auto-calibration mechanism of the MPR121 capacitive sensor to eliminate any subtle baseline capacitance changes that might have occurred during cleaning. Capacitive touch interaction was recorded via the Noldus IO box along with video using a Microsoft c930e Webcam at 800 × 600 resolution. The camera was modified to detect only infra-red (IR) light by removing the IR cut filter and placing an IR-pass filter over the lens (https://www.alcs.ch/logitech-c910-infrared-conversion-for-nightvision.html), and IR light was used to illuminate the arena. This allowed for consistent quality video, even under dim lighting conditions.

### Analysis

The CapTouch system was compared with the manual scoring using the manual scoring feature of Ethovision. During manual scoring, the scorer considered investigation of the object to begin the video frame after the animal’s nose orients toward the object within two centimeters of the object. Investigation ends the video frame that the animal’s nose moves away from the object. For both CapTouch 1.0 and 2.0, two manual scorers were used. The scorers were aware of the overall goals of the project but were trained to use the parameters for what is considered investigation described above and scored both the familiarization and test phases of the experiments. The total investigation time for both objects was summed for each session and then used to calculate a Pearson correlation between manual and CapTouch scoring for all trials. Repeated measures ANOVA was used to analyze the novel object recognition task for the CapTouch 1.0 objects. Interaction with the familiar object and novel object during the test phase were compared by calculating a percent investigation for each object. For the novel object: (novel investigation time/total time × 100); and for the familiar object: (familiar investigation time/(total time × 100)).

### Statistics

Data were analyzed using IBM SPSS statistics. A Pearson correlation was used to examine the correlation between manual scoring and CapTouch scoring for all trials and significance was evaluated with a two-tailed *t* test. Repeated measures ANOVA was used to analyze the novel object recognition task for CapTouch 1.0 objects and mean interaction time comparisons between manual and CapTouch. Statistical significance was considered at *p* < 0.05.

## Results

### CapTouch 1.0

#### Capacitive sensing validation

To test the validity of the CapTouch 1.0 system, videos from the object preference task, the familiarization phase of the 10-min delay novel object recognition task, and the test phase of the 10-min delay novel object recognition task were compared with manual scoring. Using the sum of object investigation across each session we found a high degree of correlation between manual scoring and the capacitive touch sensing (Pearson correlation, *R*^2^ = 0.9216, *p* < 0.0001; Fig. 2*H*). Looking at average object interaction for both manual scoring and capacitive touch sensing, CapTouch 1.0 has a slightly lower mean interaction duration compared with manual scoring [(± Standard Error of the Mean (SEM)) 21.11 ± 4.68 vs 26.06 ± 4.73 s, *F*_(1,51)_ = 13.816, *p* = 0.001, partial η^2^ = 0.213]. When comparing the capacitive touch sensing against an additional scorer, a strong correlation was found (Pearson correlation, *R*^2^ = 0.9167, *p* < 0.0001; Extended Data Fig. 2-1*A*), as well as when comparing the two manual scorers against each other (Pearson correlation, *R*^2^ = 0.9950, *p* < 0.0001; Extended Data Fig. 2-1*B*). Figure 2*I* provides a representative 30 s example that compares the triggering of the capacitive sensing system against manual scoring. The time between object interactions, i.e., the inter-interaction interval, was calculated and did not differ between manual and capacitive sensing (Extended Data Fig. 2-2).

#### Object preference

Figure 2*D* shows the percent investigation of the objects over 10-min trials. It was shown that there was a lack of preference between the two objects (*p* = 0.7776). The spiral and sphere objects were then used in the novel object recognition task since an innate preference was not found.

#### Novel object recognition

Results from the 10-min delay NOR task for the CapTouch 1.0 system show the mice had a significant preference for the novel object when compared with the familiar object during the test phase after a 10-min retention interval (*F*_(1,12)_ = 17.418, *p* = 0.001, effect size: partial η^2^ = 0.592; Fig. 2*E*). This shows that learning occurred during the familiarization phase and short-term memory of the familiar object was intact during the test phase. Results from the 24-h delay NOR task for the CapTouch 1.0 system also shows that mice had a significant preference for the novel object compared with the familiar object during the test phase after a 24-h retention interval (*F*_(1,19)_ = 10.615, *p* = 0.004, partial η^2^ = 0.358; Fig. 2*F*). To further examine interaction preference for the novel object compared with the familiar object, a discrimination index [(novel interaction time – familiar interaction time)/total interaction time] was calculated for the 10-min delay (*p* = 0.0013 relative to a chance score of zero) and 24-h delay (*p* = 0.0165 relative to a chance score of zero) NOR experiments (Fig. 2*G*).

### CapTouch 2.0

#### Capacitive sensing validation

To test the validity of the CapTouch 2.0 system, the videos from the familiarization and test phase of the validation experiment were manually scored for object interaction and correlated against the TTL pulses the CapTouch 2.0 system picked up from object interaction. Using this methodology, we observed a high degree of correlation between capacitive touch sensing and manual scoring (Pearson correlation, *R*^2^ = 0.9767, *p* < 0.0001; Fig. 3*D*). Similar to CapTouch 1.0, the average interaction for CapTouch 2.0 also had a slightly lower mean interaction compared with manual scoring (11.68 ± 2.95 vs 13.21 ± 3.37 s, *F*_(1,18)_ = 15.607, *p* = 0.027). A strong correlation was found when comparing the capacitive touch sensing against an additional scorer (Pearson correlation, *R*^2^ = 0.9316, *p* < 0.0001; Extended Data Fig. 3-1*A*), as well as when comparing the two manual scorers against each other (Pearson correlation, *R*^2^ = 0.9642, *p* < 0.0001; Extended Data Fig. 3-1*B*). Figure 3*E* provides a 30-s representative example that compares the triggering of the capacitive sensing system against manual scoring.

## Discussion

Here, we describe a novel approach to object recognition tasks using 3D-printed capacitive sensing which can be used to standardize the objects used and the method for scoring object investigation. Two iterations of the CapTouch system were created and tested: CapTouch 1.0 and CapTouch 2.0. In our experiments for CapTouch 1.0, the objects were 3D printed with a conductive filament that allowed for the object itself to serve as a capacitive sensor. The objects were tested against each other and no preference was found between them, which is a critical validation step when choosing objects. Basic novel object recognition tests were performed that shows the system’s accuracy compared with manual scoring and confirmed that mice were able to distinguish between the two objects, showing preference toward the novel object when introduced to it. The CapTouch 2.0 approach allows for the use of any 3D-printed filament by making the objects hollow and coating the inside with copper tape to provide the object’s conductivity. Both the CapTouch 1.0 and 2.0 approaches show a high positive correlation when compared against manual scoring from multiple scorers, indicating the CapTouch system is a reproducible and viable method regardless of the iteration used.

These experiments provide a proof-of-concept demonstration that capacitive touch sensing can be a reliable method for detecting investigation in object recognition tasks. We hope this research can pave the way for future studies to begin validating standardized object sets that can be used across labs and institutions. We validated an initial set of two object pairs, but our overall approach will allow for a concerted global effort to develop a standardized battery of objects that can be used to vary different dimensions of object properties, including size, color, and shape. The parameter space for this is quite large and will take a substantial effort to cross-validate across labs, but we feel that such an effort will be worthwhile for the field. Standardizing objects will help to reduce the current state of the field, which is characterized by a large variability in the types of objects that are used. In addition, the more standardized and high-throughput method for detecting object investigation developed here will aid in this standardization effort by reducing the personnel time required to obtain accurate data. Finally, this system can be implemented at a relatively low cost as it uses inexpensive, off-the-shelf components to easily allow labs to conduct their own studies and potentially participate in cross-validation studies.

The CapTouch system has the possibility to be versatile and modified to the user’s needs. There is noteworthy opportunity to create different object designs and choose different colors using 3D printing. Also, the sensitivity of the capacitive touch sensing can be adjusted at both the hardware and software levels, allowing the system to be more sensitive or less sensitive to interaction and scaled to work with a range of rodent sizes. This capability allows for this system to easily transition between different rodent models in object recognition assays.

### System limitations

Despite our convincing proof-of-principle demonstration, capacitive sensing does have some limitations that will be addressed in future iterations. First, it detects slightly less interaction than manual scoring does due to a greater requirement for direct physical contact. This is clearly still sufficient for conducting object recognition experiments, as we demonstrate here, but could miss brief exploratory interactions that may be particularly relevant when combining with neural recordings. The second limitation, found only in the CapTouch 1.0 model, is the use of conductive filament. This type of filament is more expensive and has limited color options compared with other types of filament. The limitation in filament colors restricts the range of available objects, so we created CapTouch 2.0 as an alternative.

The third limitation with the CapTouch 1.0 model was that once the mice became comfortable around the objects, they began climbing and sitting on them. Climbing in object recognition tasks is often debated on whether it should be considered object investigation. Climbing has been associated with significantly longer exploration, slower habituation to objects, and higher discrimination in objects (Heyser and Chemero, 2012). This alters the object investigation data because the mice are no longer investigating the objects but instead using the objects as a pedestal to gain a different vantage point of the area. To combat this limitation, CapTouch 2.0 objects were created with pointed tops to deter the mice from climbing and sitting on the objects, which may have contributed to the overall reduced object investigation between the two methods.

Perhaps the most serious limitation could be the 3D printing filament itself. Our experience suggests that the level of object investigation may be less than what we would typically expect. In hopes to increase object investigation, before running the 24-h retention interval novel object recognition experiment, the mice were exposed to objects with the same 3D-printed material as the objects that were being used in the familiarization and test phases while being handled. It seems that this exposure helped increase investigation with some mice but not all. The variability seen within a cohort of mice in the different levels of investigation could be because of the sensitivity of the mice to volatile organic compounds that off-gas from the objects. A recent report suggests this could be the case (Tropea et al., 2019); however, future work needs to be done to determine the extent to which this is, in fact, a problem and what methods can be employed to mitigate it (providing sufficient time to off-gas any aversive volatile compounds, etc.).

### Future directions

The most immediate future directions focus on addressing the limitations presented above. First, we are currently investigating ways to streamline the CapTouch system setup to make it more reliable and easier to implement. Similar to other DIY projects, we plan to support further development, implementation, and standardization experiments by hosting a web-based forum to collaboratively track progress. Our initial major goals are to make the overall system more robust to facilitate the ease of set up and take-down of the components. We plan to develop a standalone data readout system that is low-cost and does not require third-party hardware, similar to Ardesch et al. (2017). The goal would be to read-out the raw analog capacitance values so that touch and release thresholds could be tweaked off-line as needed rather than hard coded into the Arduino code. This would give us greater flexibility to titrate the sensitivity to ensure all interactions are detected. Our long-term and most ambitious goal is to spur an effort across multiple labs to develop and standardize object sets, similar to stimulus sets in human psychology (Olszanowski et al., 2014)

In conclusion, the CapTouch system presented here provides investigators with a low-cost and easily reproducible system to score object investigation in rodents. The 3D-printed object capabilities and the open-source availability of this system could be used to standardize objects used in object recognition assays across labs. Widespread use of standardized objects and methods for measuring investigation would revolutionize the use of object recognition tasks. This would ultimately lead to a better understanding of the basic mechanisms of learning and memory and substantially improve animal models of neurodegenerative and neuropsychiatric disorders overall.

## Synthesis

Reviewing Editor: Mark Laubach, American University Decisions are customarily a result of the Reviewing Editor and the peer reviewers coming together and discussing their recommendations until a consensus is reached. When revisions are invited, a fact-based synthesis statement explaining their decision and outlining what is needed to prepare a revision will be listed below. The following reviewer(s) agreed to reveal their identity: Jackson Cone.

Dear authors,

Thank you for submitting your manuscript to eNeuro. It received two positive reviews. Please see the reviews below and revise your manuscript to address all points that were raised. Your revised manuscript will be re-reviewed to ensure that all issues were addressed adequately. Please include a point-by-point reply to the reviews and be sure to address an issue raised by Reviewer 2 that you need to validate the method across longer retention intervals. Please contact me if you are not able to collect new data to address this concern. Thank you.

Regards,

Mark

Reviewer 1

In their paper, “3D Printed Capacitive Sensor Objects for Object Recognition Assays” the authors embark on the laudable goal of developing new methods for standardizing and expediting data acquisition and analysis for the widely used novel object recognition task in rodents. To do so, they design 3d printable objects and use off the shelf hardware and software to create objects with capacitive touch sensors. This approach has several key advantages: 1 - It removes data contamination that can result from variation across manually scoring observers. 2 - It eliminates the human capital cost required to manually score experimental videos. 3 - It enhances reproducibility across labs by developing standardized procedures for the novel object recognition task.

The possibility of extending this technique to include a standardized set of printable objects, each capable to automating data collection in the novel object recognition task would be very useful for the field. This paper is timely given the current push for standardization of behavioral approaches with open source tools in neuroscience, and I am fully supportive of and enthusiastic about its goal. The approach and the data are straightforward. The manuscript is easy to understand and the logic is easy to follow. Below, I raise several addressable concerns for the editors and authors to consider in a revised version.

Data Scoring:

Little is said about the scorer. Was there only 1 scorer? Was the scorer blind to the goals of the experiment? Did they know they were being pitted against automated data collection equipment in the same sessions? While the human/CapTouch data comparisons are in strong agreement, comparing against a single observer seems insufficient. At the very least, the CapTouch data should be compared to one other blind scorer. This would allow the authors to demonstrate reproducibility of their effects (by plotting a version of Figure 2F for each scorer) and potentially strengthen their argument by showing differences across scorers (by plotting a version of Figure 2F for one scorer versus the other).

Benchmarking Human versus Capacitive Scoring:

According to the methods, the human observer scored interactions based on whether the animals snout was facing the object and the animal was within 2 cm. How does this distance compare to the triggering settings used in their CapTouch sensors? Does the animal have to be physically contacting the object to trigger a TTL or are TTLs generated if the animal is similarly within the 2 cm radius used for the human observer. To facilitate comparisons, these criteria should be titrated to be as similar as possible. Can the authors speak to this or show the triggering data graphically?

Data Presentation and Analyses:

For the data points in Figure 2D-F, It needs to be made explicit where the data come from in the text and legends. For example, the methods section says that 16 mice (8F, 8M) were used in the CapTouch 1.0 experiments. In Figure 2D there are only 8 points per bar, were only 8 of the 16 mice tested for an object preference? In Figure 2E, there are 14 points per bar, were 2 of the mice excluded? The authors should clarify whether mice were excluded or not tested in each condition.

Often times in the novel object recognition task data like this are plotted as a Discrimination Ratio/Index. The data as shown are fine as is, but it might be more readily apparent to readers who used this task frequently to also plot the data using other conventions.

In Figure 2F, the scale and point size used obscures much of the data at the bottom of the plot. It appears there are many points below the x-axis for manually scored data. This makes it difficult to discern whether the manual scorer is systematically under estimating values for mice who interact less frequently with the objects compared to the CapTouch sensors. While the scoring methods are strongly correlated, were the distributions significantly different? Figure 2F could be re-plotted on a log-log scale to better resolve the lower end of the plot or the lone highest value could be excluded from the plot for the scale to better much the majority of the data. Moreover here there are many more than 16 points, so the data must come from familiarization and test sessions - this should be stated in the figure legend. Alternatively, rather than showing the data as total interaction time, the data could be plotted separately for novel versus familiar objects or as a discrimination ratio for test sessions.

For Figure 3D - same comments apply as for Figure 2F. There were 8 mice (4F, 4M) in the CapTouch 2.0 study but here there are 15 points. The legend should state the whether the data come from both familiarization and test sessions and whether all 8 mice contributed to the plot.

Did authors attempt to quantify other metrics related to behavior? For example, what about the inter-interaction interval (the time measured between subsequent interactions)? Does the CapTouch system enable researchers to capture other metrics that might be missed by a manual scorer?

Introducing CapTouch 2.0:

The rationale for conducting the CapTouch 2.0 experiments is clear and important but it comes too late in the paper. Perhaps this information could first appear as a set up paragraph in the first part of the CapTouch 2.0 section in the results. As written, the reader is left to wonder why the CapTouch 2.0 experiments were even conducted until very late in the paper.

Minor:

Fig. and Figure are used interchangeably when referring to figures and figure panels.

It would be useful to include costs in the parts list tables as well as a ballpark total cost in the text.

Reviewer 2

The current manuscript presents a new system for measuring object interaction, a common dependent variable in the novel object recognition and novel location recognition tasks. The goal is to create a cost-effective standardized stimulus set and scoring system that can be used across labs. The CapTouch system described here would be of great interest to several labs studying object memory using the novel object, novel location, or the more recently described objects in updated locations (OUL) paradigm as it would save time spent hand-scoring and provide an unbiased measurement of behavior. Further, this would bring object memory into the realm of automated behavioral scoring which has been in widespread use in other types of behavioral assays (e.g., freezing, operant lever pressing, etc.) for a considerable time.

Major Point:

1) This system needs to be validated across longer retention intervals if it is to be used widely as a standardized way of examining behavior. Right now, the use of the CapTouch system is limited to a very short (i.e., 10-min) retention interval. It is much more standard to have at least a 24-hour retention interval between the first exposure to the objects and the test phase in both novel object and novel location tasks (see Fortress et al., 2018; Kwapis et al., 2019; Pereira et al., 2014; Vogel-Ciernia & Wood, 2014, for a few examples of this).

Minor Points:

1) In Figure 2b the main comparison is within-subjects comparison therefore it is likely appropriate to calculate error bars to correct for between-subject variability (see Cousineau, 2005).

2) Please provide the raw data for hand-scoring as extended data. A positive correlation suggests that as one increases, the other also does. However, I would be interested to see if there were numerical differences between the two methods when examining the same subject (i.e., does hand-scoring suggest longer amounts of time engaging with each object than the CapTouch system when scoring the same animal?).
