# Supplementary material for: 3D-Printed Capacitive Sensor Objects for Object Recognition Assays
Source: eNeuro. 2021 Jan 25;8(1):ENEURO.0310-20.2020. doi: 10.1523/ENEURO.0310-20.2020 (PMC7877456; doi:10.1523/ENEURO.0310-20.2020)
Supplement: Extended Data Figure 2-1 — A, Correlation of object investigation duration (seconds) between capacitive touch sensing and an additional manual scorer (R2 = 0.9167, p < 0.0001). B, Comparison of manual scorers correlated against each other (R2 = 0.9950, p < 0.0001). Download Figure 2-1, PDF file. [file enu-eN-OTM-0310-20-s01.pdf]

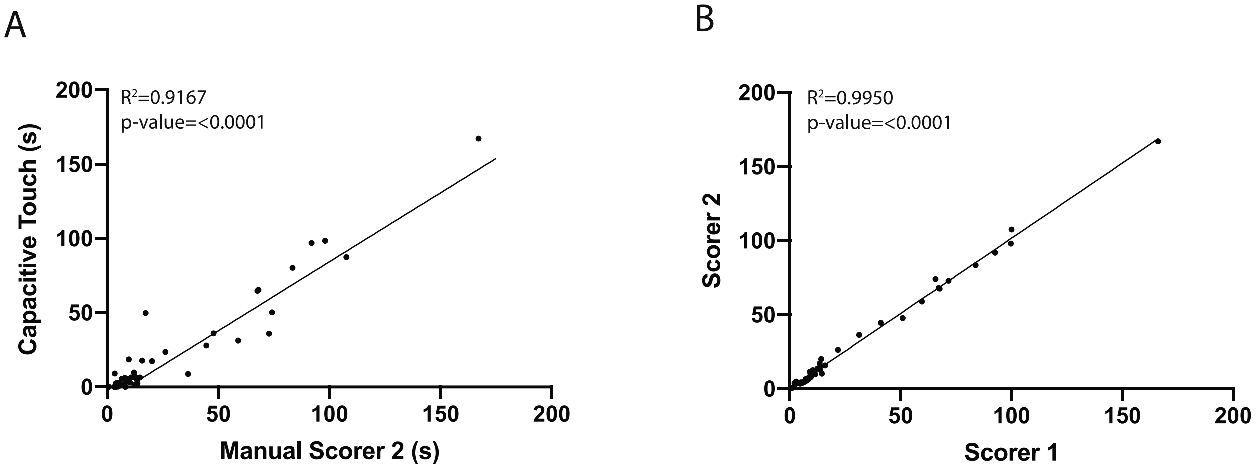

Extended Data 2-1. CapTouch 1.0 Additional Manual Scorers. A, Correlation of object investigation duration (sec) between capacitive touch sensing and an additional manual scorer (R^2=0.9167, p-value=<0.0001). B, Correlation of manual scorers against each other for CapTouch 1.0 experiments (R^2=0.9950, p-value=<0.0001).
