# Supplementary material for: 3D-Printed Capacitive Sensor Objects for Object Recognition Assays
Source: eNeuro. 2021 Jan 25;8(1):ENEURO.0310-20.2020. doi: 10.1523/ENEURO.0310-20.2020 (PMC7877456; doi:10.1523/ENEURO.0310-20.2020)
Supplement: Extended Data Figure 2-2 — A, Histogram of time between object interactions (inter-interaction interval) for capacitive sensing (mean = 13.9 ± 2.7s) and manual scoring (mean = 17.3 ± 1.7 s) for the CapTouch 1.0 experiments (i > 0.05, moody test). B, Violin plot of interinteraction intervals for capacitive sensing and manual scoring for the CapTouch 1.0 experiments. Download Figure 2-2, PDF file. [file enu-eN-OTM-0310-20-s02.pdf]

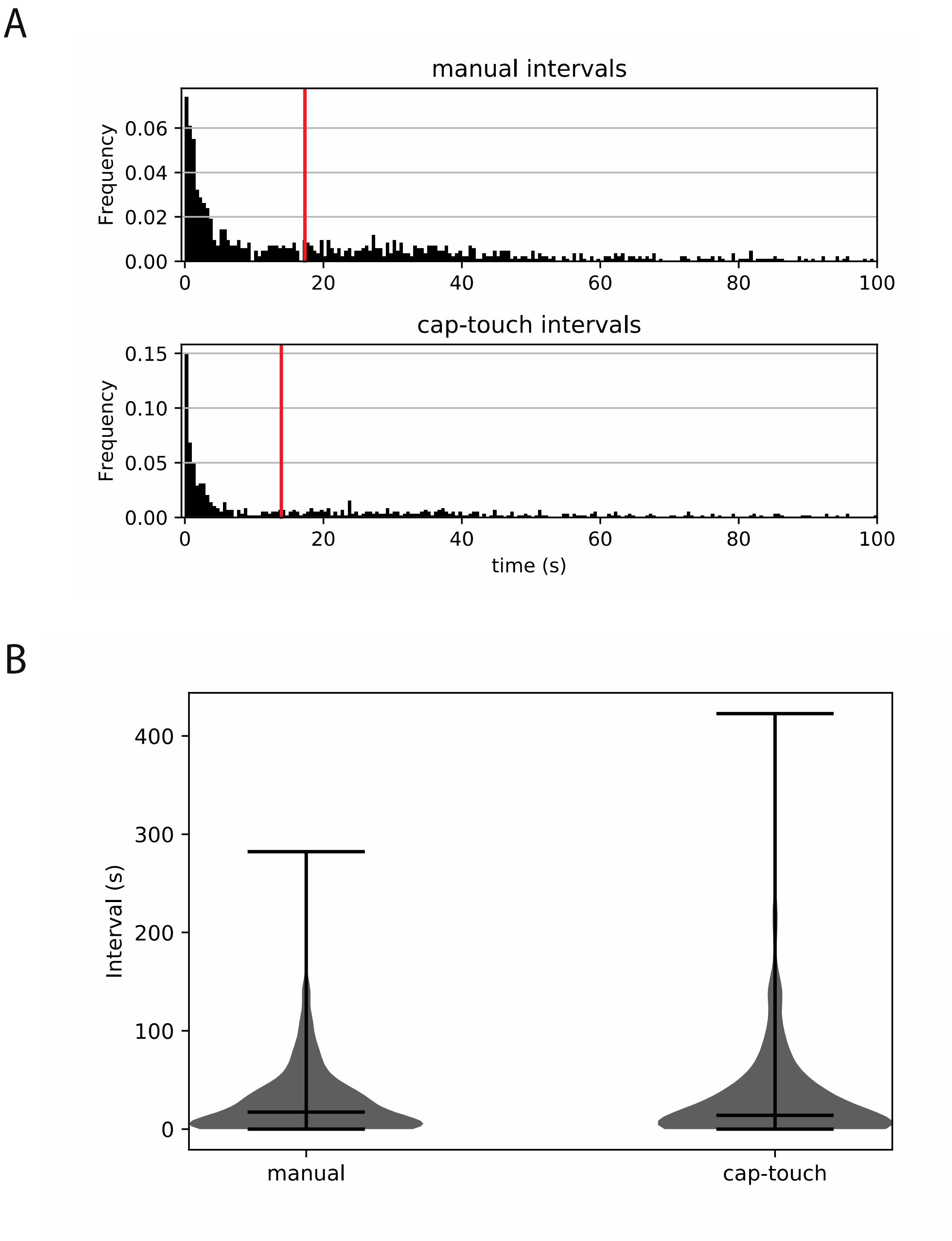

Extended Data 2-2. CapTouch 1.0 Inter-interaction interval. A, Histogram of time between object interactions for capacitive sensing (mean=13.9±2.7s) and manual scoring (17.3±1.7s) for the CapTouch 1.0 experiment ($p>0.05$, Moody test). B, Violin plot of inter-interaction intervals for capacitive sensing and manual scoring for the CapTouch 1.0 experiments.
