# Supplementary material for: 3D-Printed Capacitive Sensor Objects for Object Recognition Assays
Source: eNeuro. 2021 Jan 25;8(1):ENEURO.0310-20.2020. doi: 10.1523/ENEURO.0310-20.2020 (PMC7877456; doi:10.1523/ENEURO.0310-20.2020)
Supplement: Extended Data Figure 3-1 — A, Correlation of object investigation duration (seconds) between capacitive touch sensing and an additional manual scorer (R2 = 0.9313, p < 0.0001). B, Correlation of manual scorers against each other (R2 = 0.9642, p < 0.0001). Download Figure 3-1, PDF file. [file enu-eN-OTM-0310-20-s03.pdf]

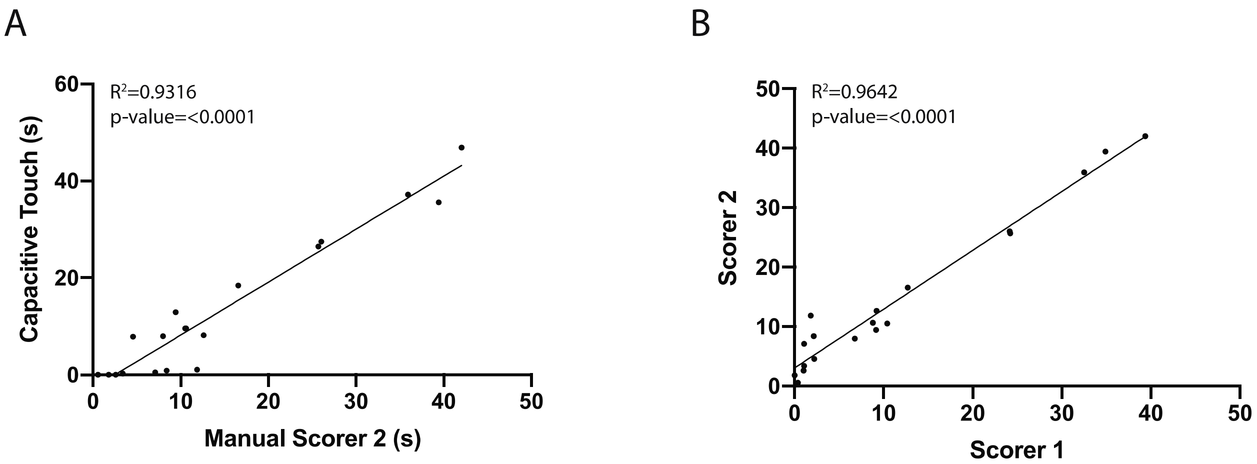

Extended Data 3-1. CapTouch 2.0 Additional Manual Scorers. A, Correlation of object investigation duration (sec) between capacitive touch sensing and an additional manual scorer (R^2=0.9316, p-value=<0.0001). B, Correlation of manual scorers against each other for CapTouch 2.0 experiments (R^2=0.9642, p-value=<0.0001).
